# Up-regulation of COX-2/PGE_2_ by endothelin-1 via MAPK-dependent NF-κB pathway in mouse brain microvascular endothelial cells

**DOI:** 10.1186/1478-811X-11-8

**Published:** 2013-01-23

**Authors:** Chih-Chung Lin, Hsi-Lung Hsieh, Ruey-Horng Shih, Pei-Ling Chi, Shin-Ei Cheng, Chuen-Mao Yang

**Affiliations:** 1 Department of Anesthetics, Chang Gung Memorial Hospital at Linkuo, and College of Medicine, Chang Gung University, Kwei-San, Tao-Yuan, Taiwan; 2 Department of Nursing, Division of Basic Medical Sciences, Chang Gung University of Science and Technology, Tao-Yuan, Taiwan; 3 Department of Pharmacology, College of Medicine, Chang Gung University, 259 Wen-Hwa 1st Road Kwei-San, Tao-Yuan, Taiwan

**Keywords:** Endothelin-1, COX-2, MAPK, NF-κB, Brain microvascular endothelial cells

## Abstract

**Background:**

Endothelin-1 (ET-1) is a proinflammatory mediator and elevated in the regions of several brain injury and inflammatory diseases. The deleterious effects of ET-1 on endothelial cells may aggravate brain inflammation mediated through the regulation of cyclooxygenase-2 (COX-2)/prostaglandin E_2_ (PGE_2_) system in various cell types. However, the signaling mechanisms underlying ET-1-induced COX-2 expression in brain microvascular endothelial cells remain unclear. Herein we investigated the effects of ET-1 in COX-2 regulation in mouse brain microvascular endothelial (bEnd.3) cells.

**Results:**

The data obtained with Western blotting, RT-PCR, and immunofluorescent staining analyses showed that ET-1-induced COX-2 expression was mediated through an ET_B_-dependent transcriptional activation. Engagement of G_i_- and G_q_-protein-coupled ET_B_ receptors by ET-1 led to phosphorylation of ERK1/2, p38 MAPK, and JNK1/2 and then activated transcription factor NF-κB. Moreover, the data of chromatin immunoprecipitation (ChIP) and promoter reporter assay demonstrated that the activated NF-κB was translocated into nucleus and bound to its corresponding binding sites in COX-2 promoter, thereby turning on COX-2 gene transcription. Finally, up-regulation of COX-2 by ET-1 promoted PGE_2_ release in these cells.

**Conclusions:**

These results suggested that in mouse bEnd.3 cells, activation of NF-κB by ET_B_-dependent MAPK cascades is essential for ET-1-induced up-regulation of COX-2/PGE_2_ system. Understanding the mechanisms of COX-2 expression and PGE_2_ release regulated by ET-1/ET_B_ system on brain microvascular endothelial cells may provide rationally therapeutic interventions for brain injury or inflammatory diseases.

## Background

Cerebral capillary and microvascular endothelial cells play an active role in maintaining cerebral blood flow, microvascular tone and blood-brain barrier (BBB) functions [1]. In the development of various vascular diseases, an early finding is dysfunction of the vascular endothelium that is closely related to clinical events in patients with atherosclerosis and hypertension [2,3]. The vasoactive mediators such as endothelin (ET) could be produced by endothelial cells to maintain hemodynamic responses. Production and release of ETs from cultured endothelial cells are regulated at transcription and translation levels by a variety of chemical and physical stimuli and the levels of ET, ET-1 especially, are elevated in shock, myocardial infarction, and kidney failure indicative of enhanced formation in these diseases [4]. Moreover, the bioactivity of ET-1 triggers vasoconstriction and pro-inflammatory action which have been implicated in the pathogenesis of hypertension and vascular diseases [3-6]. The effects of ET-1 are mediated through a G protein*-*dependent regulation, including two types of ET receptors: ET type A (ET_A_) and type B (ET_B_) [7]. ET_A_ is involved in constriction and proliferation of vascular smooth muscle cells, whereas ET_B_ on endothelial cells mediates the generation of nitric oxide, which acts as vasodilator and inhibits platelet aggregation [8]. Moreover, ET-1 also plays a substantial role in the normal development or in the central nervous system (CNS) diseases. In brain, endothelial cells [9] and astrocytes [10] are potential sources of ET-1 release in response to hypoxic/ischemic injury of the brain. A report has shown that the ET_B_ receptors are located on brain endothelial and vascular smooth muscle cells, and modulate post-injury responses of these cells in the CNS [6]. Thus, there is an increasing interest in the regulatory role of endothelial cells in neurovascular coupling, which matches adequate supply of cerebral blood flow with the local metabolic demands that are imposed by neural activity [11]. As a fundamental component of the neurovascular unit, endothelial dysfunction has been shown to be implicated in neurodegenerative diseases [11,12]. Circumstantial evidence has further demonstrated that overexpression of ET-1 on endothelial cells has deleterious effects on ischemic brain [1]. It has been demonstrated that endothelial ET-1 induces cytokines or chemokines (*e.g.*, interleukine-1 or interleukin-8) production and secretion by non-neuronal cells, including astrocytes and human brain-derived endothelial cells, which directly contributes to BBB breakdown during CNS inflammation [11,13]. These findings suggest that ET-1 might be involved in neuroinflammation. However, the detailed mechanisms responsible for ET-1 action are still limited.

Cyclooxygenase (COX), known as prostaglandin-endoperoxide synthase, is a rate-limiting key enzyme in the synthesis of prostaglandins (PGs). In this process, phospholipase A_2_ catalyzes the release of arachidonic acid (AA) from membrane phospholipids, while COX catalyzes the conversion of AA into PGs [14,15]. COX exists two isoforms: COX-1, which is constitutively expressed under normal conditions in most tissues, mediates regulating normal physiological responses and controls vascular homeostasis; COX-2, is not detectable in most normal tissues or cells, but its expression can be induced by a variety of stimuli such as cytokines, endotoxin, and growth factors to produce PGs during inflammatory responses in various cell types like vascular endothelial and smooth muscle cells [16,17]. Previous reports have shown that COX-2 immunoreactivity is a characteristic finding in the synovial macrophage and vascular cells of patients with arthritis and atherosclerosis, respectively. Moreover, several studies have indicated COX-2 as a major therapeutic target for the treatment of inflammatory disorders like arthritis [14]. The mice with homozygous deletion of the *cox-2* gene lead to a striking reduction of endotoxin-induced inflammation [18]. Accordingly, COX-2 may play a crucial role in the development of various inflammatory responses including vascular inflammation. In the CNS, several studies have indicated that up-regulation of COX-2 leads to production of PGs which are potent inflammatory mediators in neurodegenerative disorders [19].

ET-1 is known to activate ET receptors (ET_A_ or ET_B_), a heterotrimeric G protein-coupled receptor (GPCR), which stimulate multiple signaling pathways and regulate diverse cellular functions [7,20-22]. The principal mechanism underlying activation by ET-1 is mediated through ET_B_ receptors coupling G_q_ proteins, resulting in activation of phospholipase C (PLC)-β, phosphoinositide (PI) hydrolysis, and formation of inositol trisphosphate (IP_3_) and diacylglycerol, leading to Ca^2+^ increase and protein kinase C (PKC) activation [22]. Activation of a G_i_ protein-coupled ET_B_ receptor has been also shown to inhibit adenylyl cyclase activity [23]. Additionally, several studies have demonstrated that activation of G_q_ and G_i_ protein-coupled receptors via different signal pathways could activate diverse mitogen-activated protein kinases (MAPKs) [24]. It has been shown that ET-1-stimulated MAPKs activation to regulate various cellular responses including cell survival, growth, proliferation, and cellular hypertrophy in several cell types [21,25]. Several studies have suggested that up-regulation of COX-2 requires activation of MAPKs and related transcription factors in various cell types [22,26,27]. Our previous reports also demonstrate that several GPCR agonists (*e.g.* sphingosine 1-phosphate, thrombin, and bradykinin) stimulate MAPKs and NF-κB activation associated with COX-2 expression in rat VSMCs and astrocytes [28,29]. Although several pro-inflammatory mediators have been extensively confirmed to rapidly up-regulate NF-κB-dependent genes such as COX-2 and play a critical role in inflammation [29,30], the signaling mechanisms by which ET-1-induced MAPKs activation linked to COX-2 expression and PGE_2_ production are not completely defined in brain microvascular endothelial cells.

In this study, we investigated the molecular mechanisms underlying ET-1-induced COX-2 expression in mouse brain microvascular endothelial (bEnd.3) cells. These findings suggested that ET-1 induces COX-2 expression at the transcriptional and translational levels, which is mediated through the ET_B_ receptor (coupling to G_i_ and G_q_)-dependent activation of ERK1/2, p38 MAPK, JNK1/2, and NF-κB pathway, leading to PGE_2_ biosynthesis in mouse bEnd.3 cells. These results provide new insights into the mechanisms of ET-1 action which may be therapeutic value in brain inflammatory diseases.

## Results

### ET-1 induces COX-2 expression and PGE_2_ release in bEnd.3 cells

**Figure 1:**
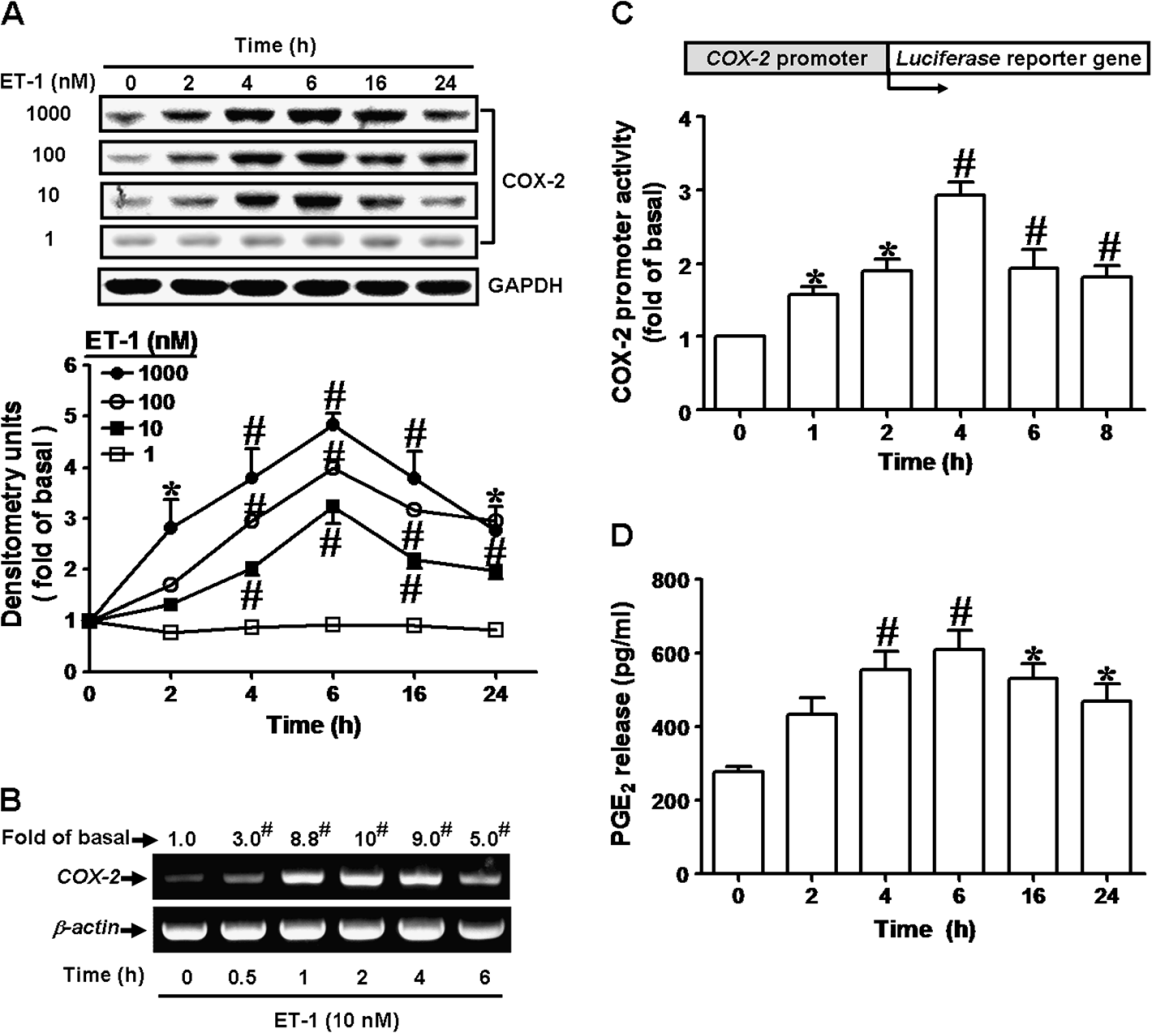
**ET-1 induced COX-2 expression and PGE_2_ release. **(**A**) Time and concentration dependence of ET-1-induced COX-2 expression, cells were treated with various concentration ET-1 for the indicated time intervals. (**B**) Time dependence of ET-1-induced COX-2 mRNA expression, cells were treated with 10 nM ET-1 for the indicated time intervals. COX-2 mRNA was analyzed by RT-PCR. (**C**) Time dependence of ET-1-induced COX-2 promoter transcription activity, cells were transfected a COX-2 promoter–luciferase reporter gene and then incubated with ET-1 for the indicated times. The promoter reporter assay was performed as described in “Materials and Methods”. (**D**) PGE_2_ release induced by ET-1, the conditioned media were collected to assay PGE_2_ level by EIA as described in “Materials and Methods” (n=3 in each group; ^*^P<0.05, ^#^*P*<0.01 compared with vehicle).

To investigate the effect of ET-1 on COX-2/PGE_2_ system, bEnd.3 cells were incubated with various concentrations of ET-1 for the indicated time intervals. The data showed that ET-1 induced COX-2 expression in a time- and concentration-dependent manner (Figure 1A). There was a significant increase within 2-4 h, reached a maximal response within 6 h, and declined within 24 h. ET-1 also time-dependently induced COX-2 mRNA expression in bEnd.3 cells, determined by RT-PCR (Figure 1B). There was a significant increase in COX-2 mRNA within 30 min, and reached a maximal response within 2 h. Moreover, to confirm whether ET-1 induces COX-2 expression via the transcription activity of COX-2 promoter, cells were transiently transfected with COX-2 promoter-luciferase reporter construct and then stimulated with ET-1 for the indicated time intervals. As shown in Figure 1C, ET-1 time-dependently induced COX-2 promoter-luciferase activity in bEnd.3 cells. A maximal response was obtained within 4 h. Our previous studies have shown that COX-2 expression induced by BK or sphingosine 1-phosphate is mainly responsible for prostanoid (PGE_2_) release in various cell types [28,29]. Thus, to determine whether ET-1 could induce PGE_2_ biosynthesis, we collected the conditioned media and determined PGE_2_ levels by using an EIA kit. The results showed that ET-1 time-dependently stimulated PGE_2_ release (Figure 1D) and a significant PGE_2_ production was observed within 4 h, reached a maximal response within 6 h and slightly declined within 24 h. These results suggested that ET-1 induces COX-2/PGE_2_ system via up-regulating COX-2 gene expression in bEnd.3 cells.

### ET-1 upregulates COX-2 expression via an ET_B_ receptor

**Figure 2:**
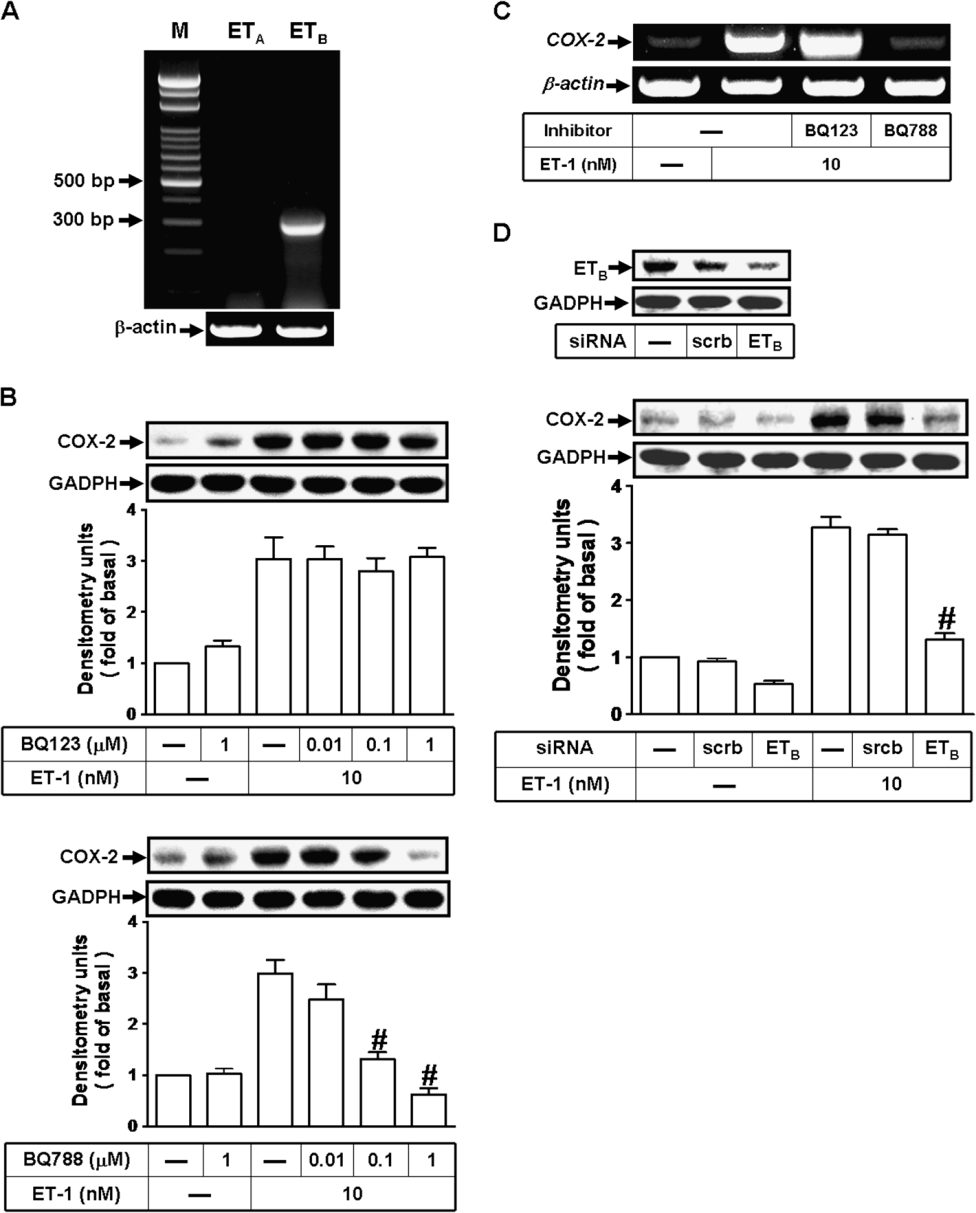
**Involvement of ET_B_ receptors in ET-1-induced COX-2 expression. **(**A**) RT-PCR analysis of ET receptor expression in bEnd.3 cells. Lane 1: maker. Lane 2: ET_A_ primers and bEnd.3 RNA. Lane 3: ET_B_ primers and bEnd.3 RNA. (**B, C**) Cells were pretreated with BQ-123 or BQ-788 for 1 h and then incubated with ET-1 for (**B**) 6 h and (**C**) 1 h. (**D**) Cells were transfected with siRNA for ET_B_ receptor for 24 h and then exposed to ET-1 for 6 h. The (**B, D**) COX-2 protein and (**C**) mRNA were analyzed by Western blot and RT-PCR, respectively as described in Figure 1. Data are expressed as mean ± SEM of three individual experiments (n=3 in each group, ^#^*P*<0.01 as compared with cells stimulated by ET-1 alone).

ET-1 exerts its biological effects via ET receptors, including ET_A_ and ET_B_, which are members of GPCR superfamily [7]. First, we determined which subtypes of ET receptors are expressed on bEnd.3 cells by RT-PCR. The data showed that ET_B_ but not ET_A_ receptors are expressed on bEnd.3 cells (Figure 2A). Next, to identify the subtypes of ET receptors involved in ET-1-induced COX-2 expression, pretreatment with BQ-788 (an ET_B_ antagonist), but not BQ-123 (an ET_A_ antagonist), attenuated the ET-1-induced COX-2 protein (Figure 2B) and mRNA (Figure 2C) expression, suggesting that ET_B_ receptor is predominantly involved in these responses. To further confirm this note, transfection of cells with ET_B_ siRNA significantly down-regulated ET_B_ protein expression and inhibited ET-1-induecd COX-2 expression (Figure 2D). These data suggested that ET-1-induced COX-2 expression is mediated through an ET_B_ receptor-dependent manner in these cells.

### Involvement of a G_i_ and G_q_ protein-coupled ET_B_ receptor in ET-1-induecd COX-2 expression

**Figure 3:**
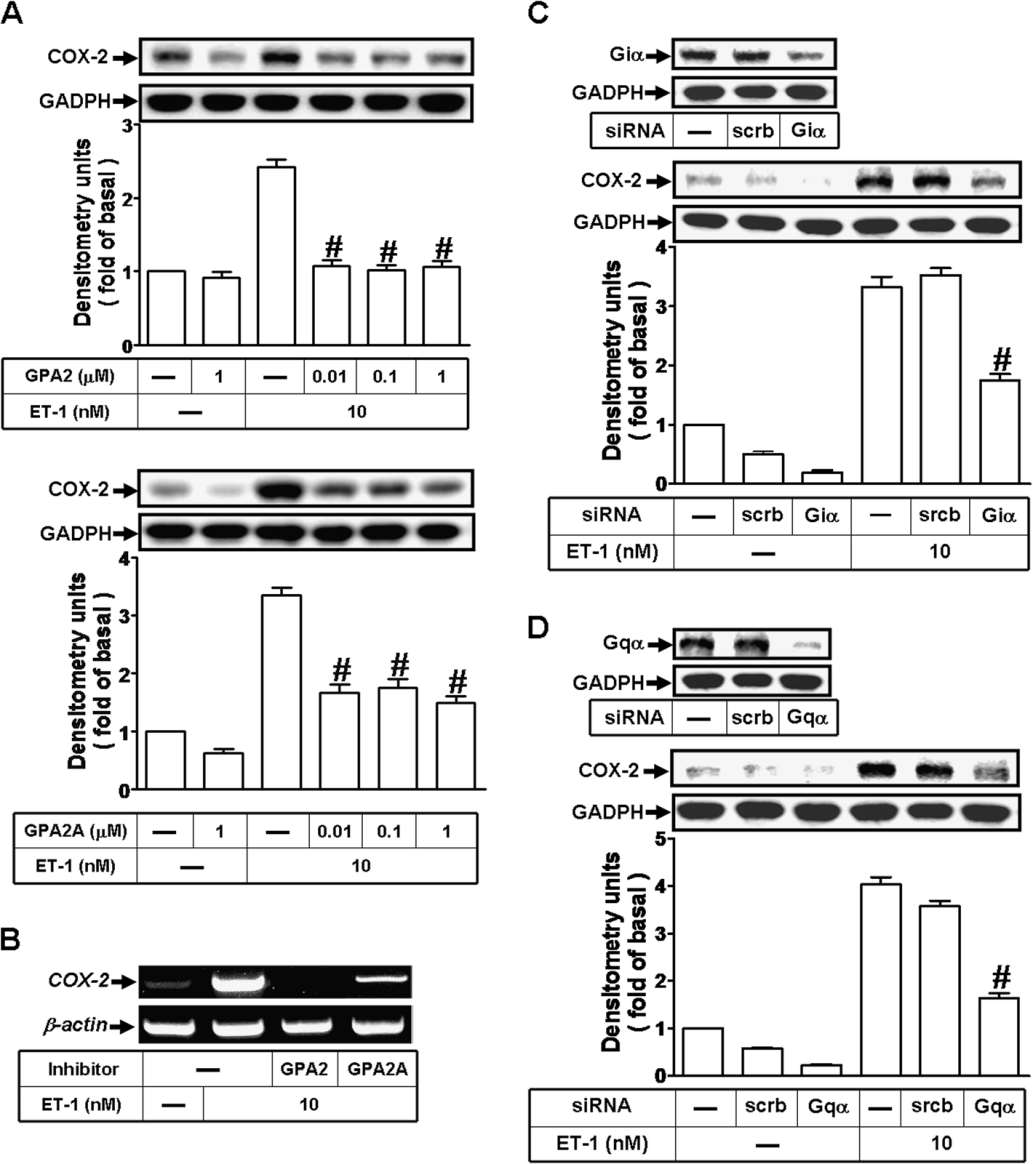
**ET-1 induces COX-2 expression via a G_i_ and G_q_ proteins-coupled ET_B_ receptor. **(**A, B**) Cells were pretreated with G_i_ antagonist (GPA2) or G_q_ antagonist (GPA2A) for 1 h and then exposed to ET-1 for (**A**) 6 h and (**B**) 1 h. (**C, D**) Cells were transfected with siRNA for (**C**) G_i_α or (**D**) G_q_α protein for 24 h and then exposed to ET-1 for 6 h. The (**A, C, D**) COX-2 protein and (**B**) mRNA were analyzed by Western blot and RT-PCR, respectively as described in Figure 1. Data are expressed as mean ± SEM of three individual experiments (n=3 in each group, ^#^*P*<0.01 as compared with ET-1 alone).

ET receptor has been shown to be a pleiotropic GPCR for ET-1 which is coupled to G proteins including G_i_ and G_q_[7,22]. To further determine which of G proteins was involved in ET-1-induced COX-2 expression, pretreatment with either G_i_ protein antagonist GP antagonist-2 (GPA2) or G_q_ protein antagonist GP antagonist-2A (GPA2A) concentration-dependently attenuated ET-1-induced COX-2 protein (Figure 3A) and mRNA (Figure 3B) expression. Furthermore, to confirm these results, as shown in Figure 3C and D, transfection with either G_i_α or G_q_α down-regulated G_i_α or G_q_α protein, respectively, and attenuated ET-1-induced COX-2 expression. These data demonstrated that ET-1-induced COX-2 expression is mediated through either G_i_ or G_q_ protein-coupled ET_B_ receptors in bEnd.3 cells.

### ET-1-induced COX-2 expression is mediated through MAPKs

**Figure 4:**
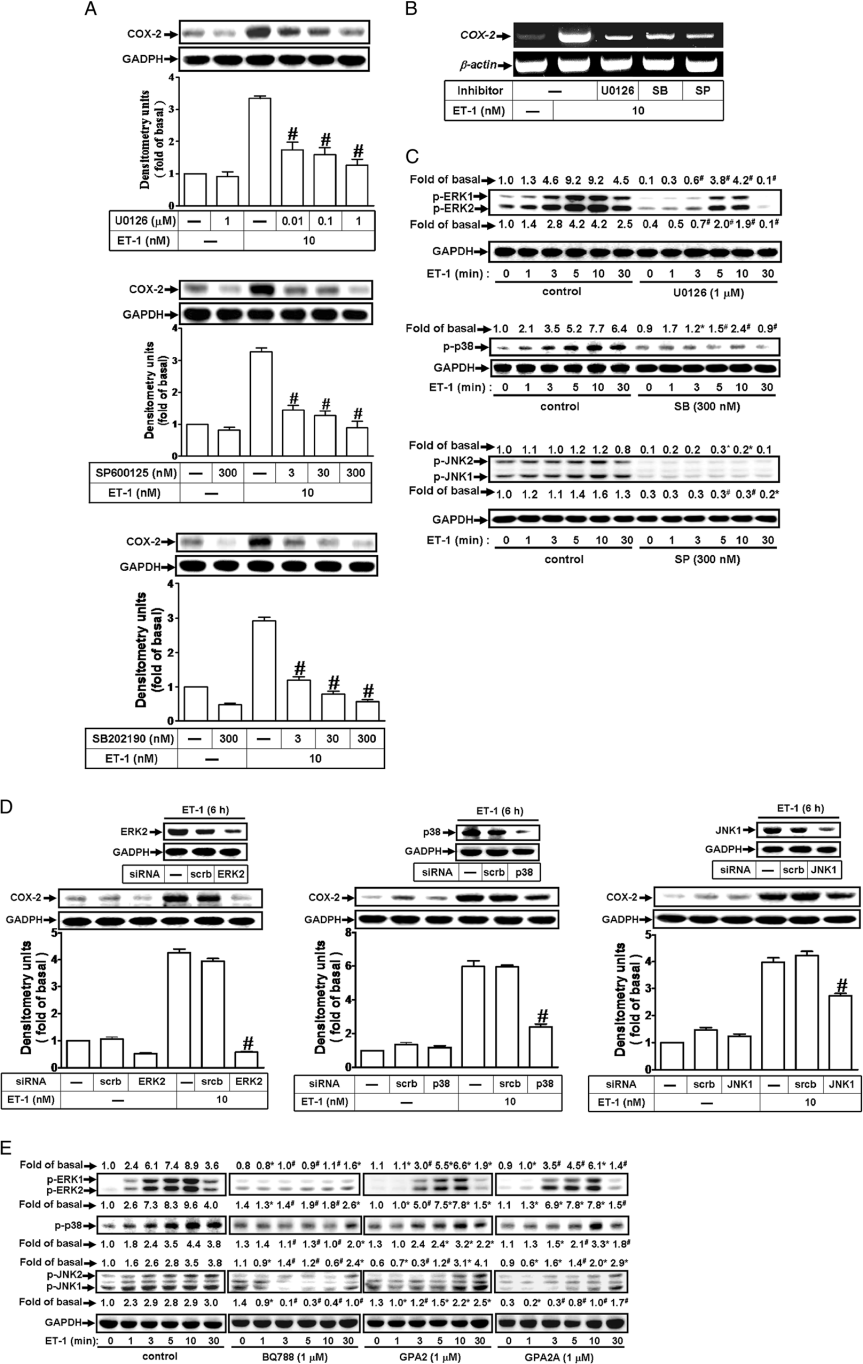
**ET-1-induced COX-2 expression is mediated through MAPKs phosphorylation.** (**A, B**) Cells were treated with 10 nM ET-1 for (**A**) 6 h and (**B**) 1 h in the absence or presence of U0126, SB202190, or SP600125. The COX-2 protein and mRNA expression were determined by Western blot and RT-PCR. (**C**) Time dependence of ET-1-stimulated ERK1/2, p38 MAPK, and JNK1/2 phosphorylation, cells were incubated with 10 nM ET-1 for the indicated times in the absence or presence of U0126 (1 μM), SB202190 (300 nM), or SP600125 (300 nM). (**D**) Cells were transfected with siRNA of ERK2, p38 MAPK, or JNK1 and then exposed to ET-1 for 6 h. (**E**) Cells were pretreated with BQ-788 (1 μM), GPA2 (1 μM), or GPA2A (1 μM) for 1 h and then incubated with ET-1 (10 nM) for the indicated times. The cell lysates were collected and analyzed by Western blotting using an anti-COX-2, anti-phospho-ERK1/2, anti-phospho-p38 MAPK, anti-phospho-JNK1/2, anti-ERK2, anti-p38 MAPK, anti-JNK1, or anti-GAPDH (as an internal control) antibody. Data are expressed as mean ± SEM of at least three individual experiments (n=3 in each group; ^*^P<0.05, ^#^*P*<0.01 as compared with ET-1 alone).

Activation of MAPKs by ET-1 could modulate cellular functions of endothelial cells [31]. To investigate the roles of ERK1/2, p38 MAPK, and JNK1/2 in ET-1-induced COX-2 expression, pretreatment with the inhibitor of MEK1/2 (U0126), p38 MAPK (SB202190), or JNK1/2 (SP600125) attenuated ET-1-induced COX-2 protein (Figure 4A) and mRNA (Figure 4B) expression in bEnd.3 cells, suggesting the involvement of ERK1/2, p38 MAPK, and JNK1/2 in ET-1-induced responses. To further determine whether ET-1-stimulated ERK1/2, p38 MAPK, and JNK1/2 phosphorylation is involved in COX-2 expression, as shown in Figure 4C, ET-1 time-dependently stimulated ERK1/2, p38 MAPK, and JNK1/2 phosphorylation which was attenuated by pretreatment with U0126, SB202190, or SP600125 during the period of observation. Moreover, to ensure the roles of MAPKs in ET-1-induced COX-2 expression, transfection with siRNA of ERK2, p38 MAPK, or JNK1 down-regulated the expression of total ERK2, p38 MAPK, or JNK1 protein and attenuated ET-1-induced COX-2 expression (Figure 4D). These data indicated that phosphorylation of ERK1/2, p38 MAPK, and JNK1/2 is involved in ET-1-induced COX-2 expression in bEnd.3 cells. To demonstrate whether ET-1 stimulates ERK1/2, p38 MAPK, and JNK1/2 phosphorylation via a G protein-coupled ET_B_ receptor cascade, pretreatment with BQ-788, GPA2, or GPA2A attenuated ET-1-stimulated ERK1/2, p38 MAPK, and JNK1/2 phosphorylation during the period of observation (Figure 4E). These results demonstrated that G_(i/q)_ protein-coupled ET_B_-dependent activation of ERK1/2, p38 MAPK, and JNK1/2 by ET-1 is, at least in part, required for COX-2 expression in bEnd.3 cells.

### NF-κB is required for ET-1-induced COX-2 expression

**Figure 5:**
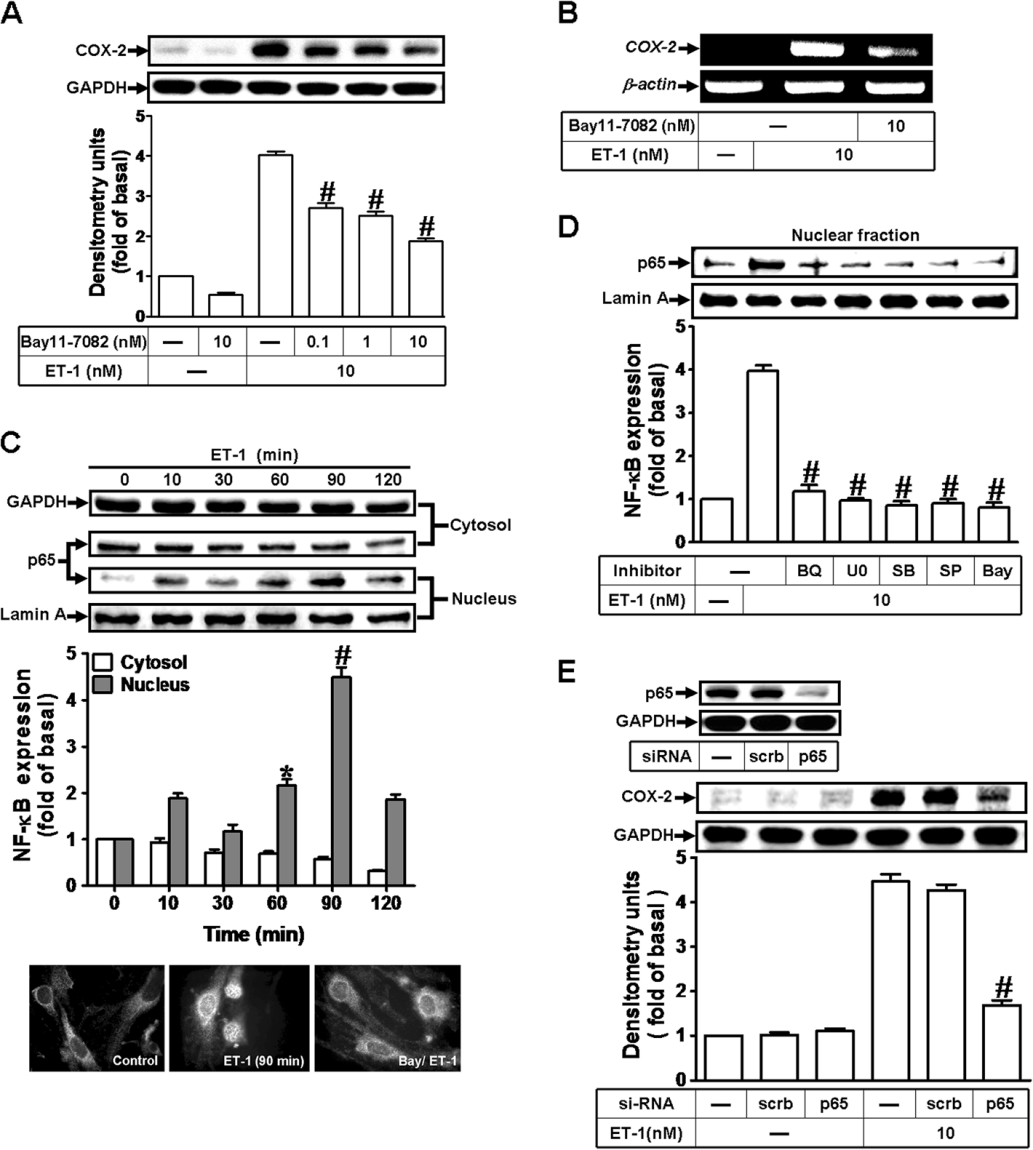
**NF-κB (p65) is essential for ET1-1-induced COX-2 expression. **(**A, B**) Cells were treated with 10 nM ET-1 for 6 h in the absence or presence of Bay11-7082. The COX-2 protein and mRNA expression were determined by Western blot and RT-PCR as described in Figure 1. (**C**) Time dependence of ET-1-stimulated p65 NF-κB translocation by subcellular isolation, Western blot, and immunofluorescent stain. (**D**) Cells were pretreated with U0126 (1 μM), SB202190 (300 nM), SP600125 (300 nM), BQ-788 (1 μM), or Bay11-7082 (10 nM) for 1 h and then incubated with ET-1 (10 nM) for 90 min. The nuclear fraction was analyzed by Western blot. (**E**) Cells were transfected with p65 siRNA and then exposed to ET-1 for 6 h. The cell lysates were collected and analyzed by Western blotting using an anti-COX-2, anti-p65, anti-Lamin A, or anti-GAPDH (as an internal control) antibody. Data are expressed as mean ± SEM of at least three individual experiments (n=3 in each group; ^#^*P*<0.01 as compared with ET-1 alone).

ET-1 has been shown to modulate cellular functions through activation of NF-κB signaling in various cell types [32]. To examine whether activation of NF-κB is required for ET-1-induced COX-2 expression, as shown in Figure 5A and B, pretreatment with a selective NF-κB inhibitor Bay11-7082, which blocks activation of NF-κB signaling, attenuated ET-1-induced COX-2 protein and mRNA expression in bEnd.3 cells. To determine whether the involvement of NF-κB in ET-1-induced responses mediated through NF-κB (p65 subunit) translocation, as shown in Figure 5C, ET-1 time-dependently stimulated translocation of NF-κB p65 from cytosol into nucleus determined by Western blot. A maximal response was obtained within 90 min and sustained over 120 min (upper part). Moreover, we also confirmed the NF-κB p65 translocation by an immunofluorescence staining. The imaging data confirmed that ET-1 stimulated the p65 translocation at 90 min, which was inhibited by pretreatment with Bay11-7082 (Figure 5C, lower part). We further demonstrated that ET-1 stimulated translocation of NF-κB p65 was attenuated by pretreatment with the inhibitor of ET_B_ receptor (BQ-788), MEK1/2 (U0126), p38 MAPK (SB202190), JNK1/2 (SP600125), or NF-κB (Bay11-7082) (Figure 5D). To further verify that NF-κB p65 is essential for ET-1-induced COX-2 expression, as shown in Figure 5E, transfection with p65 siRNA significantly reduced the p65 protein expression (upper panel) and the ET-1-induced COX-2 expression (lower panel). The results suggested that ET-1-stimulated NF-κB translocation mediated through ET_B_ receptor, ERK1/2, p38 MAPK, and JNK1/2 is required for COX-2 induction in bEnd.3 cells.

### Involvement of NF-κB in COX-2 gene promoter activity stimulated by ET-1

**Figure 6:**
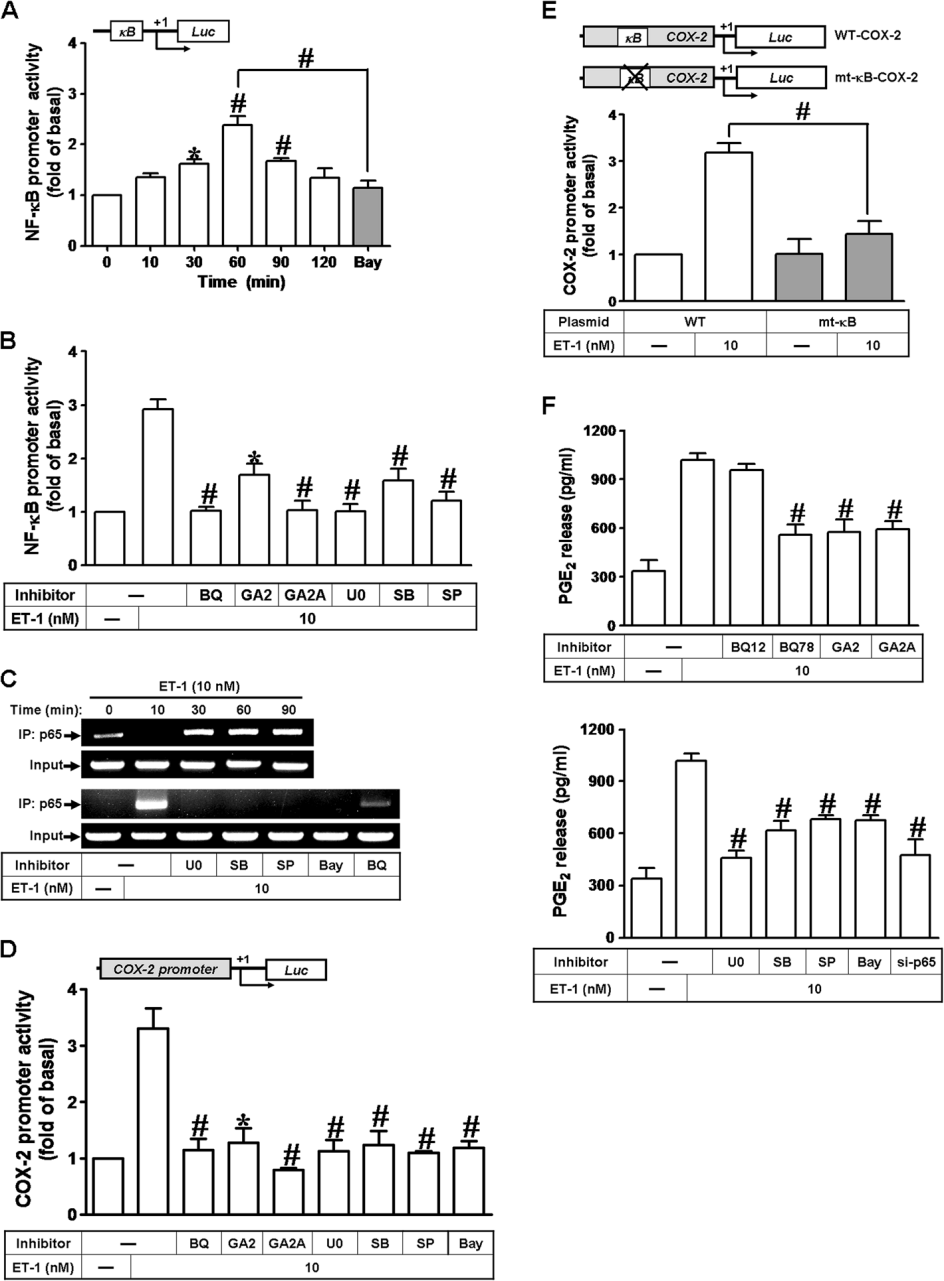
**ET-1-stimulated COX-2 promoter activity is mediated through NF-κB-dependent pathway. **(**A**) Time dependence of ET-1-enhanced NF-κB transcription activity, cells were transfected with a NF-κB-luciferase reporter gene and then exposed to ET-1 for the indicated times. (**B**) After transfection, the cells were pretreated with BQ-788 (1 μM), GPA2 (1 μM), GPA2A (1 μM), U0126 (1 μM), SB202190 (300 nM), SP600125 (300 nM), or (**A**) Bay11-7082 (10 nM) for 1 h and then incubated with ET-1 (10 nM) for 60 min. (**C**) Cells were pretreated with BQ-788, U0126, SB202190, or SP600125 for 1 h and then incubated with ET-1. The p65 NF-κB binding activity was analyzed by ChIP-PCR as described in Methods. (**D**) For COX-2 promoter activity, cells were transfected with a COX-2-promoter-luciferase reporter gene and then exposed to ET-1. After transfection, the cells were pretreated with BQ-788, GPA2, GPA2A, U0126, SB202190, SP600125, or Bay11-7082 for 1 h and then incubated with ET-1 for 6 h. (**E**) Schematic representation of a 5′-promoter regions of the mouse different COX-2 promoter constructs, either wild-type (WT) or mutation of the κB-binding site (mt-κB) fused to the pGL-luciferase reporter gene, the translational start site (+1) of the luciferase reporter gene was indicated by an arrow. Cells were transfected with WT COX-2 promoter reporter gene (WT-COX-2) or NF-κB mutated COX-2 promoter reporter gene (mt-κB-COX-2) and then incubated with or without ET-1 for 6 h. The promoter reporter assay was performed as described in Methods. (**F**) Cells were pretreated with BQ-123, BQ-788, GPA2, GPA2A, U0126, SB202190, SP600125, Bay11-7082, or transfected with p65 siRNA and then incubated with ET-1 for 6 h. The PGE_2_ levels were analyzed by EIA. Data are expressed as mean ± SEM of at least three individual experiments (n=3 in each group; ^#^*P*<0.01 as compared with ET-1 alone).

We have found that ET-1 stimulates translocation of NF-κB p65 leading to COX-2 expression (Figure 5). Next, we examined whether activation of NF-κB is essential for ET-1-induced COX-2 gene up-regulation. The transcriptional activity of NF-κB was evaluated by a promoter (containing NF-κB binding sites)-luciferase activity assay. As shown in Figure 6A, ET-1 enhanced NF-κB transcriptional activity in a time-dependent manner with a maximal response within 60 min, which was significantly inhibited by pretreatment with an inhibitor of NF-κB (Bay11-7082, 10 nM). Moreover, pretreatment with BQ-788 (1 μM), GPA2 (1 μM), GPA2A (1 μM), U0126 (U0, 1 μM), SB202190 (SB, 300 nM), or SP600125 (SP, 300 nM) attenuated NF-κB transcriptional activity stimulated by ET-1 (Figure 6B), demonstrating that ET-1 enhances the NF-κB transcriptional activity through an ET_B_-dependent activation of MAPKs. Subsequently, we determined that ET-1-stimulates NF-κB p65 binding activity in a time-dependent manner by ChIP-PCR analysis (Figure 6C). ET-1-stimulated NF-κB p65 binding activity was inhibited by pretreatment with U0126, SB202190, SP600125, Bay11-7082, or BQ-788 (Figure 6C, lower part). In addition, we have demonstrated that ET-1 time-dependently induces COX-2 promoter activity (Figure 1C). We further demonstrated that ET-1-increased the COX-2 promoter activity was significantly inhibited by pretreatment with BQ-788, GPA2, GPA2A, U0126, SB202190, SP600125, or Bay11-7082 (Figure 6D), suggesting that ET-1 stimulates COX-2 promoter activity via the ET_B_-dependent activation of MAPKs and NF-κB in bEnd.3 cells. To further ensure that NF-κB indeed mediates ET-1-induced COX-2 promoter activity through binding to its regulatory element within the COX-2 promoter region, the wild-type (WT) and mutated by a single-point mutation of the NF-κB binding site (mt-κB) COX-2 promoters were constructed (as illustrated in Figure 6E, upper panel). ET-1-stimulated COX-2 promoter activity was significantly attenuated in bEnd.3 cells transfected with mt-κB-COX-2 (Figure 6D, lower panel), indicating that NF-κB element was essential for ET-1-induced COX-2 promoter activity. These results further confirmed that ET-1 induces COX-2 promoter activity via enhancing NF-κB binding to the κB binging site within COX-2 promoter region in bEnd.3 cells.

We have found that ET-1 time-dependently induces PGE_2_ release (Figure 1D). Here, we further determined the involvement of these signaling components in ET-1-induced PGE_2_ release, as shown in Figure 6F, ET-1-induced PGE_2_ release was markedly attenuated by pretreatment with BQ-788, GPA2, GPA2A, U0126, SB202190, SP600125, Bay11-7082, or transfection with p65 siRNA. These results demonstrated that ET_B_-mediated activation of MAPKs (*i.e.,* ERK, p38 MAPK, and JNK) and NF-κB by ET-1 is essential for COX-2 up-regulation and PGE_2_ release in bEnd.3 cells.

## Discussion

Several lines of evidence have demonstrated that high levels of PGs, synthesized by inducible COX-2, are involved in inflammatory responses. The up-regulation of COX-2 has been shown to display a wide range of biological activities in different tissues, including development, proliferation, cancers, and inflammation [14,15]. Moreover, ET-1 is elevated in the regions of vascular injuries and inflammation [7,8]. Circumstantial evidence has further demonstrated that overexpression of ET-1 on endothelial cells has deleterious effects on ischemic brain [1,5,6]. Reid et al. (1995) suggest that the ET-1 model provides new insights into the mechanisms of cerebral ischemia and reperfusion injury, and evaluates the usefulness of novel strategies of neuroprotection [33]. ET-1 has been shown to up-regulate the expression of COX-2 through MAPKs in various cell types [26,27,34]. However, little is known about the effect of ET-1 on COX-2 expression in brain vascular endothelial cells. Here, we applied cultured models of mouse bEnd.3 cells coupled with Western blot analysis, selective pharmacological inhibitors, transfection with siRNAs, immunofluorescenct staining, and promoter assay to investigate the molecular mechanisms underlying ET-1-induced COX-2 expression and PGE_2_ release. Our results demonstrate that in bEnd.3 cells, activation of ET_B_ receptor-dependent MAPKs (ERK1/2, p38, and JNK1/2) and NF-κB signaling cascade is essential for ET-1-induced COX-2 gene expression and PGE_2_ release.

ET-1 activates ET receptor subtypes (ET_A_ and ET_B_) which are coupled to various G proteins such as G_q_ and G_i_ and then lead to multiple signaling pathways and regulate diverse cellular functions [7,20-22]. Thus, we first demonstrated a significant expression of ET_B_ receptor in mouse bEnd.3 cells (Figure 2A). The involvement of ET_B_ receptors in these responses is confirmed by that pretreatment with BQ-788 (an ET_B_ receptor antagonist) reduced the ET-1-induced COX-2 protein and mRNA expression (Figure 2B and 2C), promoter activity (Figure 6D), and PGE_2_ release (Figure 6F), but not by an ET_A_ receptor antagonist BQ-123. Subsequently, we confirmed these results by transfection with ET_B_ siRNA (Figure 2D), suggesting that ET_B_ receptor predominantly mediates ET-1-induced COX-2 expression and PGE_2_ release in bEnd.3 cells. Next, several subtypes of G proteins are potentially implicated in ET-1-induced COX-2 expression. We use GPA2 (a G_i_ protein antagonist) and GPA2A (a G_q_ protein antagonist) to interrupt G protein signaling and consequent COX-2 expression (Figure 3A). Moreover, the inhibitory effects of GPA2 and GPA2A on COX-2 induction by ET-1 were also observed in its mRNA (Figure 3B), promoter activity (Figure 6D), and PGE_2_ release (Figure 6F), indicating that ET-1-induced COX-2 expression and PGE_2_ release is mediated through a GPCR (*i.e.* ET_B_) coupling to either G_i_ or G_q_ protein in bEnd.3 cells, consistent with previous studies from esophageal smooth muscle cells [34] and rat brain astrocytes [22]. In contrast, previous reports have shown that ET-1 induces COX-2 expression via ET_A_ receptors in peripheral lung microvascular smooth muscle cells [25] and ET-1 (ET_A_) receptors linked to phospholipase C and phospholipase A_2_ activation and prostanoid secretion (*e.g.* PGE_2_) in cultured human brain microvascular endothelial cells [35,36]. However, in respiratory and cardiovascular systems, both ET receptor subtypes, ET_A_ in particular, are involved in progression of several diseases [37,38]. There differences may be due to cell type specific or different experimental conditions.

Abnormal MAPK regulation might be implicated in several models of CNS injury and inflammation [39]. Several lines of evidence demonstrate that MAPKs could be activated by GPCR agonists through different signaling pathways [24]. MAPKs activation by ET-1 has been shown to modulate various cellular responses in several cell types [22,25]. Activation of ERK1/2 (p44/p42 MAPK) might be implicated in the expression of inflammatory genes in several models of vascular injury and inflammation [17,28]. In this study, we demonstrated that ET-1 stimulated an ET_B_ receptor-dependent cascade of sequential ERK1/2 phosphorylation (Figure 4E), which contributes to induction of COX-2 protein and mRNA levels (Figure 4A and 4B), promoter activity (Figure 6D), and PGE_2_ release (Figure 6F). The involvement of ERK1/2 in COX-2 expression and PGE_2_ release was furthe confirmed by transfection of cells with p42 siRNA (Figure 4D). These results are consistent with those of obtained with COX-2 expression induced by BK, thrombin, or ET-1 in various cell types [17,26,28]. Additionally, we found that expression of COX-2 and release of PGE_2_ induced by ET-1 were also attenuated by the inhibitor of p38 MAPK or JNK1/2. Pretreatment with SB202190 or SP600125 both markedly reduced ET-1-induced expression of COX-2 protein and mRNA (Figure 4A and 4B), promoter activity (Figure 6D), and PGE_2_ release (Figure 6F). Moreover, we also demonstrated that ET-1 stimulates phosphorylation of p38 MAPK and JNK via an ET_B_-dependent manner (Figure 4C and 4E). Similarly, we further confirmed these results by transfection with siRNA for p38 MAPK or JNK1 that attenuated ET-1-induced COX-2 expression (Figure 4D). These data clearly indicated that in bEnd.3 cells, three MAPK cascades (*i.e.* ERK1/2, p38 MAPK, and JNK1/2) are required for ET-1-induced COX-2 expression and PGE_2_ release. These results are consistent with those of obtained with up-regulation of COX-2 by ET-1 via p38 MAPK in glomerular mesangial cells or esophageal smooth muscle cells [27,34]. For the role of JNK1/2, we are the first presented that JNK1/2 plays a critical role in induction of COX-2 by ET-1 in endothelial (bEnd.3) cells.

It has been well established that inflammatory responses following exposure to extracellular stimuli are highly dependent on activation of NF-κB transcription factor, which plays an important role in regulation of several gene expression [40]. The 5’-flanking region of the COX-2 promoter has been shown to contain several binding sequences for various transcription factors including NF-κB [41]. Therefore, the regulation of COX-2 transcription may be mediated by aberrant activation of several distinct transcription factors dependent on agonists [29,42]. These reports suggest that NF-κB plays a critical role in the regulation of COX-2 expression in the development of the inflammatory responses. Our data showed that ET-1-induced COX-2 gene expression and PGE_2_ release was significantly abolished by a selective NF-κB inhibitor Bay11-7082 (Figures 5 and 6) or NF-κB p65 siRNA (Figures 5E and 6F), suggesting that NF-κB (p65) is involved in ET-1-induced COX-2 expression in bEnd.3 cells. Moreover, ET-1-stimulated NF-κB p65 translocation (Figure 5C), binding to COX-2 promoter region (Figure 6C and 6E), and NF-κB transcriptional activity (Figure 6A) was significantly inhibited by Bay11-7082 and the MAPK inhibitor U0126 (MEK1/2), SB202190 (p38 MAPK), or SP600125 (JNK1/2) (Figures 5 and 6). Our data further showed that ET-1-stimulated NF-κB transcriptional activity was significantly attenuated by blocking G_i_ and G_q_ protein-coupled ET_B_ receptor-dependent pathways (Figure 6B), indicating that ET-1-induced activation of NF-κB is mediated through ET_B_ receptor-dependent activation of three MAPKs cascades. These findings are consistent with recent studies indicating that COX-2 expression and prostacyclin release induced by thrombin were mediated through MAPKs and NF-κB activation in endothelial cells [16] and vascular smooth muscle cells [17] and COX-2 expression and PGE_2_ release induced by BK via ERK1/2 linking to NF-κB activation in astrocytes [29]. The involvement of NF-κB in ET-1-induced COX-2 expression is also consistent with previous reports indicating that ET-1-stimulated activation of NF-κB regulates expression of target genes involved in various CNS inflammatory processes [22]. Moreover, our recent data have also demonstrated that in bEnd.3 cells, c-Src-dependent transactivation of EGFR/PI3K/Akt and MAPKs linking to c-Jun/AP-1 cascade is essential for ET-1-induced COX-2/PGE_2_ upregulation [43]. We suggest that the findings of these two studies might have a crosstalk in MAPKs and lead to COX-2 expression induced by ET-1 in these cells. The interplay between these two pathways in the induction of COX-2 will be investigated in the future.

## Conclusions

In this study, we reported here that ET-1/ET (ET_B_) receptor system exerts its effects on COX-2 gene expression and PGE_2_ release in mouse bEnd.3 cells. The G_i_ and G_q_ protein-coupled ET_B_ receptor, ERK1/2, p38 MAPK, JNK1/2, and NF-κB cascades cooperatively mediated these effects of ET-1. These findings concerning ET-1-induced COX-2/PGE_2_ system imply that ET-1 might play a critical role in brain injury, vascular inflammation, and CNS diseases, mediated through MAPK-dependent activation of NF-κB pathway in bEnd.3 cells. Pharmacological approaches suggest that targeting COX-2/PGE_2_ system and their upstream signaling components should yield useful therapeutic targets for brain injury and inflammatory diseases.

## Methods

### Materials

Dulbecco’s modified Eagle’s medium (DMEM)/F-12 medium, fetal bovine serum (FBS), and TRIzol were from Invitrogen (Carlsbad, CA). Hybond C membrane and enhanced chemiluminescence (ECL) Western blot detection system were from GE Healthcare Biosciences (Buckinghamshire, UK). Anti-COX-2 monoclonal antibody was from BD Transduction Laboratories (San Diego, CA). Phospho-ERK1/2, phospho-p38, phospho-JNK1/2 antibody kits were from Cell Signaling (Danver, MA). p65, p42 (ERK2), p38, and JNK1 antibodies were from Santa Cruz (Santa Cruz, CA). Anti-glyceraldehyde-3-phosphate dehydrogenase (GAPDH) antibody was from Biogenesis (Boumemouth, UK). BQ-123, BQ-788, GP antagonist-2, GP antagonist-2A, U0126, SB202190, SP600125, and Bay11-7082 were from Biomol (Plymouth Meeting, PA). Bicinchoninic acid (BCA) protein assay reagent was from Pierce (Rockford, IL). ET-1, enzymes, and other chemicals were from Sigma (St. Louis, MO).

### Mouse brain microvascular endothelial cell culture

Mouse brain microvascular endothelial cells (bEnd.3) were purchased from Bioresource Collection and Research Centre (BCRC, Hsinchu, Taiwan) and grew in DMEM/F-12 containing 10% FBS and antibiotics (100 U/ml penicillin G, 100 μg/ml streptomycin and 250 ng/ml fungizone) at 37°C in a humidified 5% CO_2_ atmosphere. The cell line is acquired from mouse BALB/c strain brain cerebral cortex endothelial polyoma middle T antigen transformed, which was performed STR-PCR profile at BCRC. All the experiments were performed using this cell line and approved by the ethic approval of Chang Gung University. Confluencent cells were released with 0.05% (w/v) trypsin/0.53 mM EDTA for 5 min at 37°C. The cell suspension (2 × 10^5^ cells/ml) was plated onto 6-well culture plates (2 ml/well) or 10-cm culture dishes (10 ml/dish) for the measurement of protein or RNA expression, respectively. Culture medium was changed after 24 h and then every 3 days. Experiments were performed with cells from passages 5 to 13.

### Preparation of cell extracts and Western blot analysis

Growth-arrested cells were incubated with ET-1 at 37°C for various time intervals. The cells were washed with ice-cold phosphate-buffered saline (PBS), scraped, and collected by centrifugation at 45,000 × g for 1 h at 4°C to yield the whole cell extract, as previously described [17]. Samples were analyzed by Western blot, transferred to nitrocellulose membrane, and then incubated overnight using an anti-COX-2, phospho-ERK1/2, phospho-p38 MAPK, phospho-JNK1/2, p42, p38, JNK1, p65, or GAPDH antibody. Membranes were washed with TTBS four times for 5 min each, incubated with a 1:2000 dilution of anti-rabbit horseradish peroxidase antibody for 1 h. The immunoreactive bands were detected by ECL reagents.

### Total RNA extraction and gene expression

For reverse transcription PCR (RT-PCR) analysis, total RNA was extracted from mouse brain endothelial cells stimulated by ET-1, as previously described [17]. The cDNA obtained from 0.5 μg total RNA was used as a template for PCR amplification. Oligonucleotide primers were designed based on Genbank entries for mouse COX-2 and β-actin. The following primers were used for amplification reaction: for COX-2: 5^′^-(AAAACCGTGGGGAATGTATGAGC)-3^′^ (sense), 5^′^-(GATGGGTGAAGTGCTGGGGAAAG)-3^′^ (anti-sense); ET_A_: 5^′^-(GGCGCAATCGCTGACAATGCTGAG)-3^′^ (sense), 5^′^-(CCACGTAGATAAGGTCTCCAAGGG)-3^′^ (anti-sense); T_B_: 5^′^-(CGTGTTCGTGCTAGGCATCATCGG)-3^′^ (sense), 5^′^-(CGACTCCAAGAAGCAACAGCTCGA)-3^′^ (anti-sense); for β-actin: 5^′^-(GAACCCTAAGGCCACCGTG)-3^′^ (sense), 5^′^-(TGGCATAGAGGTCTTTACGG)-3^′^ (anti-sense). PCR mixes contained 10 μl of 5X PCR buffer, 1.25 mM of each dNTP, 100 pmol of each forward and reverse primer, and 2.5 units of Taq polymerase (Takara, Shiga, Japan). The final reaction volume was 50 μl. Amplification was performed in 25 cycles at 94°C, 20 s; 60°C, 40 s; 72°C, 40 s [44]. After the last cycle, all samples were incubated for an additional 10 min at 72°C. PCR fragments were analyzed on 2% agarose 1X TAE gel containing ethidium bromide and their size was compared to a molecular weight marker. Amplification of β-actin, a relatively invariant internal reference RNA, was performed in parallel, and cDNA amounts were standardized to equivalent β-actin mRNA levels. These primer sets specifically recognized only the genes of interest as indicated by amplification of a single band of the expected size (500 bp for COX-2, 343 bp for ET_A_; 293 bp for ET_B_, and 514 bp for β-actin) and direct sequence analysis of the PCR products.

### Immunofluorescence staining

Cells were plated on 6-well culture plates with coverslips. Cells were shifted to a serum-free DMEM/F-12 for 24 h and treated with 10 nM ET-1. After washing twice with ice-cold PBS, the cells were fixed with 4% (w/v) paraformaldehyde in PBS for 30 min, and then permeabilized with 0.3% Triton X-100 in PBS for 15 min. The staining was performed by incubating with 10% normal goat serum in PBS for 30 min followed by incubating with a primary anti-p65 NF-κB polyclonal antibody (1:200 dilution) for 1 h in PBS with 1% BSA, washing thrice with PBS, incubating for 1 h with fluorescein isothiocyanate (FITC)-conjugated goat anti-rabbit antibody (1:200 dilution) in PBS with 1% BSA, washing thrice with PBS, and finally mounting with aqueous mounting medium. The images observed under a fluorescence microscope (ZEISS, Axiovert 200M).

### Chromatin immunoprecipitation assay

To detect the *in vivo* association of nuclear proteins with mouse COX-2 promoter, chromatin immunoprecipitation (ChIP) analysis was conducted as previously described [45]. Briefly, the bEnd.3 cells were cross-linked with 1% formaldehyde for 10 min at 37°C and washed thrice with ice-cold PBS containing 1 mM phenylmethylsulfonyl fluoride (PMSF) and 1% aprotinin. Soluble chromatin was prepared using a ChIP assay kit (Upstate) according to the manufacturer’s recommendations and immunoprecipitated without (control) or with anti-p65 NF-κB antibody and normal goat immunoglobulin G (IgG). Following washes and elution, precipitates were heated overnight at 65°C to reverse cross-linking of DNA and protein. DNA fragments were purified by phenol-chloroform extraction and ethanol precipitation. The purified DNA was subjected to PCR amplification using the primers specific for the region (-371 to +70) containing the NF-κB binding site present in the COX-2 promoter region, sense primer: 5^′^-GGGGGAGGGAAGCTGTGACACTCTTGAGCTTT-3^′^; antisense primer: 5^′^-GACAGTGCTGAGATTCTTCGTGAGCAGAGTCC-3^′^. PCR fragments were analyzed on 2% agarose in 1X TAE gel containing ethidium bromide and the size (441 bp) was compared to a molecular weight marker.

### Plasmid construction, transient transfection and luciferase assays

The mouse COX-2 promoter was constructed as described previously [46] with some modifications. The upstream region (-907 to +70) of the mouse COX-2 promoter was cloned to the pGL3-basic vector containing the luciferase reporter system. Introduction of a double-point mutation into the NF-κB-binding site (TGGGGA to TGGGAC) to generate pGL-COX2-mκB was performed using the following (forward) primer: 5^′^-GGGGTACCGCAAATAATTTTTTATCAAACACTGTTTCTG-3^′^ (corresponding to a region from -907 to +70). The underlined nucleotides indicate the positions of substituted bases. The mutant construct was cloned into the pGL3-basic vector containing the luciferase reporter system. All plasmids were prepared by using QIAGEN plasmid DNA preparation kits. The siRNAs for p42, p38, JNK1, p65, and scrambled control were from Dharmacon Research Inc (Lafayette, CO), and NF-κB or COX-2 promoter constructs were transfected into cells using the Lipofetamine-2000 transfection reagent according to the instructions of manufacture (Invitrogen, Carlsbad, CA). The transfection efficiency (~60%) was determined by transfection with enhanced EGFP. To assess promoter activity, cells were collected and disrupted by sonication in lysis buffer (25 mM Tris-phosphate, pH 7.8, 2 mM EDTA, 1% Triton X-100, and 10% glycerol). After centrifugation, aliquots of the supernatants were tested for luciferase activity using a luciferase assay system. Firefly luciferase activities were standardized to β-galactosidase activity.

### Measurement of PGE_2_ release

The cells were seeded in 12-well plates and grown to confluence. Cells were shifted to serum-free DMEM/F-12 medium for 24 h, and then treated with ET-1 for various time intervals. The culture supernatants were collected to measure PGE_2_ levels using an EIA kit as specified by the manufacturer (Cayman Chemical).

### Statistical analysis of data

All data were estimated using GraphPad Prism Program (GraphPad, San Diego, CA). Quantitative data were analyzed by one-way ANOVA followed by Tukey’s honestly significant difference tests between individual groups. Data were expressed as mean±SEM. A value of *P*<0.05 was considered significant.

## Abbreviations

CNS: Central nervous system; BBB: Blood-brain barrier; ET-1: Endothelin-1; COX-2: Cyclooxygenase-2; PGE_2_: Prostaglandin E_2_; bEnd.3: Brain microvascular endothelial cells; DMEM/F-12: Dulbecco’s modified Eagle’s medium/Ham’s nutrient mixture F-12; FBS: Fetal bovine serum; ECL: Enhanced chemiluminescence; BCA: Bicinchoninic acid; PBS: Phosphate-buffered saline; GPCR: G protein-coupled receptor; siRNA: Small interfering RNA; RT-PCR: Reverse transcription-polymerase chain reaction; ChIP: Chromatin immunoprecipitation.

## Competing interests

The authors have no competing interests to declare.

## Authors’ contributions

CCL, RHS, PLC, and SEC designed and performed experiments, acquisition and analysis of data, and drafted the manuscript. HLH and RHS helped to perform experiments and prepare the manuscript. HLH and CMY have conceived of the study, participated in its design and coordination, CMY has been involved in drafting the manuscript and revising it critically for important intellectual content and has given final approval of the version to be published. All authors read and approved the final manuscript.
